# Lumbar Spine Mucormycosis Mimicking Pott’s Spine: A Case Report

**DOI:** 10.1155/crdi/1299422

**Published:** 2026-06-08

**Authors:** Kanav Gupta, Kashif Qureshi, Sonal Gupta, Rakesh Dua, Shivanya Singh, Shaurya Darbari, Bipin Chaurasia, Kivanc Yangi

**Affiliations:** ^1^ Department of Neurosurgery, All India Institute of Medical Sciences (AIIMS), Jammu, India, aiims.edu; ^2^ Department of Neurosurgery, Yale School of Medicine, New Haven, Connecticut, USA, yale.edu; ^3^ Department of Neurosurgery, Fortis Hospital, Shalimar Bagh, New Delhi, India, fortishealthcare.com; ^4^ Department of Oncoanesthesia and Palliative Care, All India Institute of Medical Sciences (AIIMS), Bhopal, India, aiims.edu; ^5^ Department of Neurosurgery, Neurosurgery Clinic, Birgunj, 44200, Nepal; ^6^ Department of Neurosurgery, Barrow Neurological Institute, Phoenix, Arizona, USA, thebarrow.org

**Keywords:** antifungal therapy, case report, immunocompromised host, mucormycosis, spinal infection, spondylodiscitis

## Abstract

**Background:**

Spinal mucormycosis is an exceptionally rare form of invasive fungal infection, with fewer than 12 confirmed cases reported in the prior literature. Immunocompromised patients are disproportionately affected, and mortality exceeds 50% in this population. Chronic liver disease as a primary predisposing condition is underrepresented in the existing case series.

**Case Presentation:**

A 54‐year‐old male with Child–Pugh Grade C chronic liver cirrhosis, diabetes mellitus, and hypothyroidism presented with progressive low back pain and bilateral lower limb weakness of one week duration. Lumbar MRI demonstrated L2‐L3 spondylodiscitis with epidural abscess formation, initially attributed to Pott’s spine, and antitubercular therapy (ATT) was initiated empirically. Rapid neurological deterioration prompted surgical re‐evaluation. The patient underwent an L3 laminectomy with drainage of an L3‐L4 anterior epidural abscess. Intraoperative microscopy revealed aseptate ribbon‐like hyphae consistent with Mucorales species. Liposomal Amphotericin B (5 mg/kg/day), intravenous micafungin, and oral posaconazole were commenced. Antifungal therapy was complicated by progressive nephrotoxicity and hepatic decompensation requiring repeated dose modifications. The patient developed hepatic encephalopathy with sepsis and died on Postoperative day (POD) 12 from multiorgan failure.

**Conclusion:**

This case represents the 12th documented instance of spinal mucormycosis and the first attributable to Child–Pugh Grade C liver disease. In tuberculosis‐endemic settings, mucormycosis must be considered in the differential diagnosis of apparent Pott’s spine in immunocompromised patients, particularly those with progressive neurological deterioration despite empirical ATT. Concurrent hepatic and renal impairment substantially constrains antifungal options and worsens prognosis. Multidisciplinary coordination and early tissue diagnosis are critical to the management of this rare, life‐threatening condition.

## 1. Introduction

Mucormycosis comprises a group of invasive fungal infections caused by saprophytic molds of the order Mucorales, encompassing genera including *Rhizopus*, *Mucor*, *Lichtheimia*, and *Cunninghamella*. Once regarded as almost exclusively a disease of diabetic ketoacidosis and hematological malignancy, the at‐risk population has broadened considerably to include patients receiving prolonged corticosteroid therapy, solid organ transplant recipients, those with end‐stage liver or renal disease, and patients recovering from critical COVID‐19 illness [1, 2]. Mortality across published series consistently exceeds 50%, and outcomes correlate closely with the anatomical site of involvement, the speed of diagnosis, and the degree of host immunosuppression [2, 3].

Among the possible anatomical manifestations, spinal mucormycosis is rare. In contrast to rhinocerebral and pulmonary forms, which together account for the majority of reported infections, vertebral and paraspinal disease has been documented in fewer than 12 cases worldwide prior to the current report [4–8]. The challenge of distinguishing rare fungal pathogens from more common diagnoses on imaging has been documented across anatomical sites, including skull base lesions that mimic malignancy [9] and spinal epidural infections caused by *Aspergillus* species [10]. The extreme rarity of spinal mucormycosis has precluded the development of evidence‐based management guidelines, and clinicians must rely on individual case experiences and principles extrapolated from treatment of more common manifestations. Mortality in immunocompromised patients with spinal involvement exceeds 50%, and rapid clinical deterioration is a near‐universal feature [8].

Geographic variation in mucormycosis incidence is substantial. Published registry data indicate annual incidence figures well below 1 case per million population in most European and North American settings, while rates in South Asia are orders of magnitude higher, driven by the combined burden of poorly controlled diabetes mellitus and increasing immunosuppression from varied clinical sources [3, 4]. In India specifically, the intersection of high tuberculosis endemicity with elevated mucormycosis incidence creates a diagnostic environment of particular hazard: both conditions produce spondylodiscitis with vertebral body destruction and paraspinal abscess on MRI, and the two are often radiologically indistinguishable [8, 11].

We report a case of lumbar spondylodiscitis caused by Mucorales in a 54‐year‐old male with Child–Pugh Grade C chronic liver disease, presenting with clinical and radiological features initially indistinguishable from Pott’s spine. The case represents the 12th confirmed instance of spinal mucormycosis in the literature and is the first to arise in the context of advanced hepatic cirrhosis as the primary immunocompromising condition. We describe the diagnostic course, surgical and antifungal management, and the specific challenges posed by concurrent hepatorenal dysfunction.

## 2. Case Presentation

**FIGURE 1:**
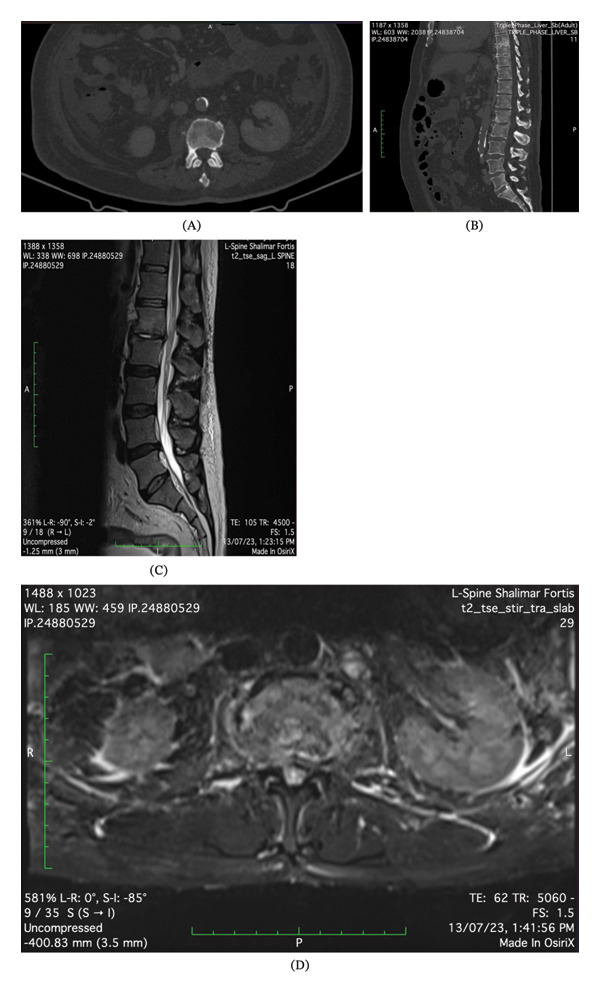
Initial CT and MRI images. (A, B) Noncontrast CT, axial and sagittal planes, demonstrating the involved level. (C) T2‐weighted sagittal MRI. (D) Axial contrast‐enhanced MRI showing disc and adjacent end plate involvement.

A 54‐year‐old male presented to the emergency orthopedics department with mechanical low back pain of one week′s duration. He was a known case of chronic liver cirrhosis with portal hypertension and ascites (Child–Pugh Grade C), diabetes mellitus, hypothyroidism, and thrombocytopenia, and had previously undergone treatment for Grade 3 esophageal varices in June 2023. No relevant family history was obtained. There was no history of trauma. MRI of the lumbar spine was obtained (Figure 1), demonstrating L2‐L3 discitis without neural compression, with features consistent with suspected Pott’s spine. Following evaluation by the gastroenterology team, antitubercular therapy (ATT) was initiated at weight‐adjusted doses.

**FIGURE 2:**
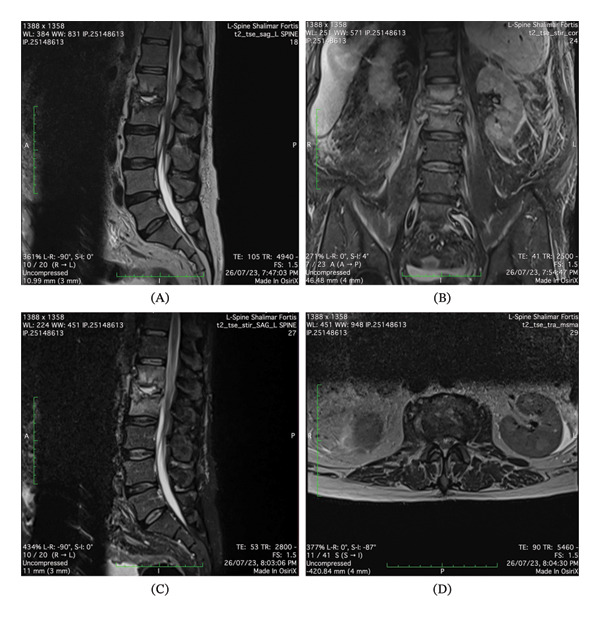
MRI images obtained after the onset of neurological deficit. (A) T2 sagittal and (B) T2 coronal images demonstrating increased signal hyperintensity along the end plates and increased cord compression relative to initial imaging. (C) Sagittal and (D) axial T1 contrast images showing increased contrast enhancement with end plate destruction and progressive neural compression.

Within one week of initial diagnosis, the patient became bed‐bound due to progressive paraparesis (Grade 3+) and was readmitted. At readmission, he reported progressive mechanical low back pain with radiation to the right lower limb, bilateral lower limb weakness, and rest pain without constitutional symptoms. On examination, there was tenderness over the lower dorsal and lumbar vertebrae. Neurological examination demonstrated 3/5 power in the left lower limb and 4/5 power in the right lower limb, with absent rectal tone. Urinary retention necessitated Foley catheter insertion. Repeat MRI of the lumbar spine (Figure 2) demonstrated spondylodiscitis with an epidural abscess, confirming worsening and progression of disease.

Radiological features included T2 hyperintensity and T1 signal change with L2‐L3 marrow edema, vertebral body and disc space destruction, and an associated epidural collection consistent with abscess. The patient was afebrile throughout. CT‐guided biopsy was considered by the interventional team; however, given the low expected tissue yield relative to the degree of neurological deficit and the clinically significant progression, the patient was planned for surgical decompression.

**FIGURE 3:**
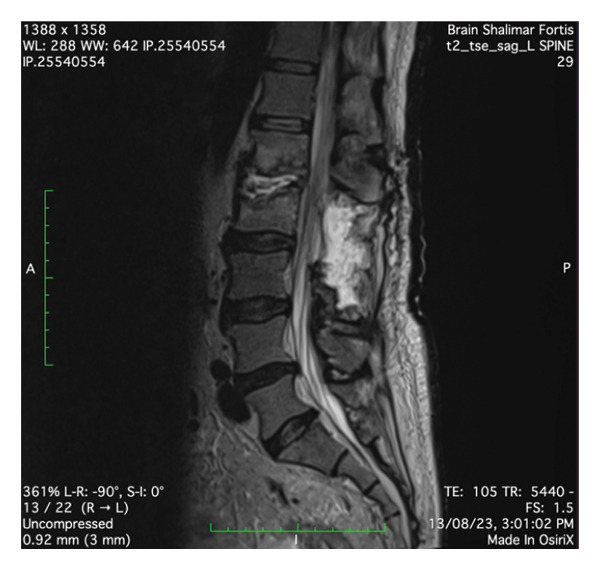
Postoperative T2‐weighted MRI images following L3 laminectomy and drainage of the L3‐L4 anterior epidural abscess.

In view of multiple comorbidities and worsening neurological status, two options were discussed with the family in a multidisciplinary team meeting. Option A: open surgical decompression under general anesthesia, carrying a recognized risk of hepatic decompensation and encephalopathy given Child–Pugh Grade C cirrhosis. Option B: less invasive CT‐guided biopsy, which would not provide cauda equina decompression and would therefore not address the neurological deficit. Immediately before the planned procedure, the patient’s right lower limb power declined acutely from 4/5 to 2/5. Following appropriate optimization, multidisciplinary clearance, and informed family consent, the patient underwent L3 laminectomy with microscopic drainage of the L3‐L4 anterior epidural abscess and tissue biopsy (Figure 3). Intraoperatively, the pus was macroscopically indistinguishable from tubercular pus. The patient was transferred to the neurosurgical ICU for strict neuromonitoring and postoperative care.

**FIGURE 4:**
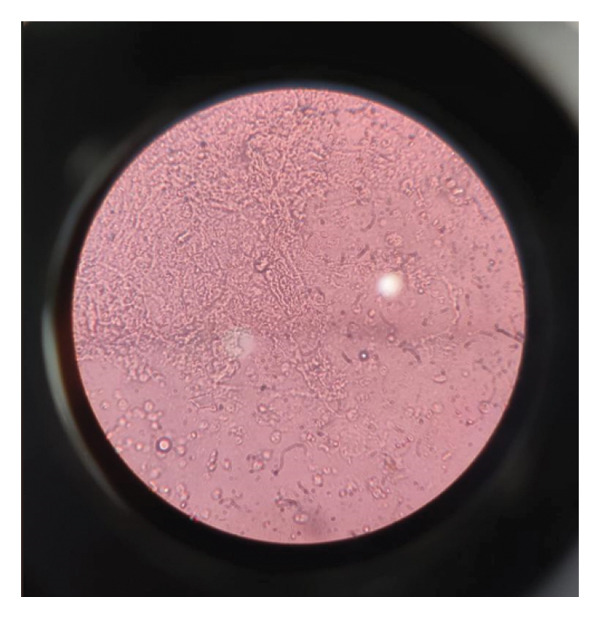
KOH mount of intraoperative pus specimen demonstrating broad, aseptate hyphae consistent with Mucorales species.

Microscopic examination of the debrided tissue revealed aseptate ribbon‐like fungal hyphae consistent with Mucorales species (Figure 4). Real‐time PCR was submitted. Liposomal Amphotericin B (5 mg/kg/day IV), intravenous micafungin, and oral posaconazole were commenced, with serum electrolyte monitoring initiated. The patient demonstrated gradual improvement in bilateral lower‐limb power during the initial postoperative period.

On Postoperative day (POD) 3, derangement of renal function with concurrent hypomagnesemia was identified. Amphotericin B was withheld, and posaconazole was continued as the sole antifungal agent. Following three days of posaconazole monotherapy, renal function stabilized, and Amphotericin B was restarted at 5 mg/kg/day. Progressive elevation of serum bilirubin and worsening ascites subsequently indicated hepatic decompensation, necessitating further withholding of Amphotericin B. The gastroenterology team performed paracentesis. The patient subsequently developed hepatic encephalopathy with sepsis, followed by acute respiratory distress. Mechanical ventilation was recommended; the patient’s family declined consent. The patient was managed according to ICU protocol and died on POD 12 from multiorgan failure. Clinical timeline from initial presentation to death on POD 12 has been summarized in Table 1.

**TABLE 1:** Clinical timeline from initial presentation to death on POD 12.

Event	Timepoint	Details
Initial orthopedic presentation	Admission (Day 0)	Mechanical low back pain, 1‐week duration; no neurological deficit; MRI: L2‐L3 discitis without cord compression, attributed to suspected Pott’s spine; antitubercular therapy initiated
First MRI lumbar spine	Day 0	L2‐L3 discitis; no neural compression; T2 signal change consistent with discitis; reported as suspected Pott’s spine
Antitubercular therapy started	Day 0–7	Weight‐optimized ATT commenced; patient discharged initially
Readmission: neurological deterioration	∼Day 7	Progressive bilateral lower limb weakness: 3/5 left, 4/5 right; urinary retention; absent rectal tone; Foley catheter inserted
Second MRI lumbar spine	Day of readmission	Spondylodiscitis with L3‐L4 epidural abscess; end plate destruction; confirmed disease progression. Laboratory indices (CRP, ESR, WBC, and serum creatinine) not available for formal documentation
Multidisciplinary team meeting	Preoperative	Option A: open decompression under general anesthesia (risk: hepatic decompensation, encephalopathy). Option B: CT‐guided biopsy (insufficient for cauda equina decompression). Option A: selected after informed family consent
Neurological deterioration (right limb)	Immediate preoperative	Right lower limb power declined acutely from 4/5 to 2/5; emergency surgical decision expedited
L3 laminectomy + L3‐L4 epidural abscess drainage	POD 0	Microscopic drainage; pus macroscopically indistinguishable from tubercular pus; tissue biopsy obtained; patient transferred to neurosurgical ICU
Histopathology result	POD ∼ 1‐2	KOH mount and microscopy: aseptate ribbon‐like hyphae consistent with Mucorales species (Figure 4); real‐time PCR submitted
Antifungal therapy commenced	POD ∼ 1‐2	Liposomal Amphotericin B 5 mg/kg/day IV; intravenous micafungin; oral posaconazole commenced; serum electrolytes monitored
Renal dysfunction and hypomagnesemia	POD 3	Serum creatinine elevated (value not recorded); hypomagnesemia confirmed; Amphotericin B withheld; oral posaconazole continued as the sole agent
Amphotericin B restarted	POD ∼ 6	Renal function stabilized after 3 days of posaconazole monotherapy; Amphotericin B resumed at 5 mg/kg/day
Progressive hepatic decompensation	POD ∼ 7‐10	Progressive bilirubin elevation (values not recorded); worsening ascites; paracentesis performed; hepatic encephalopathy with sepsis developed; Amphotericin B withheld again; posaconazole continued
Respiratory distress; family decision	POD ∼ 11	Acute respiratory distress, labored breathing; mechanical ventilation advised; family declined consent; ICU protocol management continued
Death	POD 12	Multiorgan failure: hepatic encephalopathy, sepsis, acute respiratory failure

## 3. Discussion

Mucorales are ubiquitous environmental molds that, under conditions of impaired host immunity, germinate and invade tissue through angioinvasion, driving the characteristic necrosis and hemorrhagic infarction observed on histopathology [1, 5]. The defining microscopic features are broad, aseptate, or pauciseptate hyphae that branch at right angles, a pattern that distinguishes Mucorales from *Aspergillus*, which produces narrow, septate hyphae at acute angles [5]. This histological distinction carries direct therapeutic implications: azole antifungals with anti‐*Aspergillus* activity, including voriconazole, lack meaningful activity against Mucorales and have been associated with clinical failure when used empirically for mucormycosis [1]. In the present case, intraoperative tissue microscopy provided the definitive diagnosis, and this was the only route available given the radiological nonspecificity and the urgency imposed by neurological deterioration.

This case represents the 12th reported instance of spinal mucormycosis, extending a literature that spans from Buruma et al. in 1979 to Patel et al. in 2022 (Table 2) [6–8, 11–14]. Among the 12 cases, 10 occurred in immunocompromised hosts. Six of these 10 immunocompromised patients died (60%), consistent with population‐level mucormycosis registry data [2, 3]. Rapid clinical deterioration is a near‐universal feature: four of the six deaths occurred within two to three weeks of admission despite antifungal therapy [7, 8, 11, 12], while Buruma et al. reported death on Day 1 of admission following diagnosis at autopsy [13], and the present patient died on POD 12. Surviving immunocompromised patients carried residual neurological deficits or evidence of persistent disease at follow‐up. Both immunocompetent patients reported in the literature survived, and in each case, a discrete iatrogenic or traumatic inoculating event was identified [6, 14]. The present patient is the first in this series in whom advanced hepatic cirrhosis was the sole predisposing condition.

**TABLE 2:** Literature review of all 12 reported cases of spinal mucormycosis, including the present case.

Study	Age/Sex	Presentation	Level	Treatment	Species	Outcome
Buruma et al., 1979	60/M	Resected cervical LN carcinoma; bilateral neck XRT; radiation ulcer; tracheostomy	C1‐C5	None	Not reported	Expired (diagnosed on autopsy)
Rozich et al., 1988	52/M	Splenectomy; MDS on chemotherapy; cauda equina syndrome	L2‐L4	L3‐L5 laminectomy; IV Amphotericin B x12 days	Not reported	Expired, POD 16
von Pohle et al., 1996	43/M	Diabetic ketoacidosis; paraplegia progressing to quadriparesis	T4‐T8	IV Amphotericin B 1 mg/kg/day x2 days	Not reported	Expired, POD 2
Chen et al., 2006	57/F	Recent L4/5 radiofrequency nucleoplasty; paraparesis	L4‐L5	L4‐L5 laminectomy with repeated debridements; IV Amphotericin B; local Amphotericin B; oral flucytosine; itraconazole	*R. rhizopodoformis*	Alive; residual abscess on follow‐up at 1 year
Skiada et al., 2009	2/M	AML on chemotherapy; status epilepticus; quadriparesis	T12‐L1	IV Amphotericin B; voriconazole; posaconazole	A. corymbifera	Alive; persistent vegetative state (concurrent intracerebral disease)
Tintelnot and Nitsche, 1989	49/M	C6 fracture‐subluxation; 1 week postfusion; wound infection	C7‐T1	IV Amphotericin B; local wound irrigation; local Amphotericin B instillation	*R. oligosporus*	Alive; no evidence of disease at 6 months
Giuliani et al., 2010	54/F	DM; cutaneous lesions; paraplegia	T10‐T12	Surgical debridement; IV amphotericin B	*R. arrhizus*	Alive at 2 years; disease status unclear
Navanukroh et al., 2014	42/F	CKD; renal transplant on immunosuppression; left lower limb radiculopathy	L4‐S1	L4‐S1 laminectomy; IV Amphotericin B; posaconazole	*C. bertholletiae*	Alive with persistent disease at last follow‐up
Hadgaonkar et al., 2015	64/M	DM; HTN; CKD; low back pain	L4‐L5	IV Amphotericin B	Not reported	Expired at 3 weeks
Shah and Nene, 2017	54/M	Liver cirrhosis; portal HTN; pancytopenia; low back pain; right leg sciatica	L3‐L4	IV Amphotericin B 5 mg/kg/day	Not reported	Expired at 2 weeks
Patel et al., 2022	36/F	T‐ALL on chemotherapy; paraparesis; T6 sensory level	T2‐T6	IV amphotericin B 7.5 mg/kg/day; IV micafungin 100 mg/day; oral posaconazole 300 mg/day x12 weeks; maintenance posaconazole	Not reported	Alive at 8 months; persistent epidural collection at 4 months
Present case (Gupta et al.)	54/M	Chronic liver disease (Child–Pugh Grade C); DM; hypothyroidism; thrombocytopenia; portal HTN; ascites	L2‐L4	L3 laminectomy + L3‐L4 epidural abscess drainage; liposomal Amphotericin B 5 mg/kg/day IV; IV micafungin; oral posaconazole	Mucorales (aseptate hyphae; species not further characterized)	Expired, POD 12 (multiorgan failure)

*Note:* Columns: case study, age and sex, comorbidities and presenting symptoms, spinal level, treatment administered, causative species (where identified), and clinical outcome. ATT, antitubercular therapy; F, female; HTN, hypertension; IV, intravenous; M, male; MDS, myelodysplastic syndrome; POD, postoperative day; T‐ALL, T‐cell acute lymphoblastic leukemia; XRT, radiotherapy.

Abbreviations: AML, acute myeloid leukemia; CKD, chronic kidney disease; DM, diabetes mellitus; ICU, intensive care unit; MIC, minimum inhibitory concentration.

The most clinically significant implication of this case relates to the diagnostic challenge in tuberculosis‐endemic settings. India carries one of the highest burdens of both tuberculosis and mucormycosis globally, and spinal tuberculosis remains the commonest cause of infectious spondylodiscitis in this context [4]. The radiological appearance in the present case prompted empirical ATT, a reasonable first‐line decision. The critical point is that the patient did not stabilize; he deteriorated neurologically despite one week of treatment. In immunocompromised patients with apparent Pott’s spine who fail to respond to ATT or who decline neurologically while on treatment, urgent tissue diagnosis must be pursued regardless of procedural risk. CT‐guided biopsy was considered and deferred in this case, given the high hepatic operative risk and the inadequacy of biopsy alone to address cauda equina compromise. The surgical decision to proceed with a laminectomy served the dual purpose of neural decompression and tissue procurement, an approach worth highlighting as a model for similar high‐risk presentations.

Antifungal management of mucormycosis centers on liposomal Amphotericin B, which offers superior tissue penetration and a more acceptable nephrotoxicity profile relative to conventional Amphotericin B deoxycholate [1]. Current international guidelines recommend doses of 5–10 mg/kg/day, continued until clinical and radiological disease stabilization is confirmed [1]. The present case illustrates the compounding difficulty of delivering adequate antifungal therapy in a patient with concurrent hepatic and renal impairment. Nephrotoxicity required suspension of Amphotericin B on POD 3; progressive hepatic decompensation subsequently limited tolerance of all agents and precluded resumption of a full‐dose course. The total antifungal exposure was less than 12 days, almost certainly insufficient for disease control in spinal mucormycosis [6]. Minimum inhibitory concentration (MIC) testing was not performed, an acknowledged limitation; susceptibility data would have been valuable in guiding agent selection given the constraints imposed by organ dysfunction. Combination therapy incorporating echinocandins alongside Amphotericin B has been reported in prior cases with theoretical synergy based on cell wall disruption, though no randomized trial evidence exists [5, 6].

Several broader lessons emerge from this case. The multidisciplinary model was not merely preferable but necessary: neurosurgery, gastroenterology, and intensive care each contributed essential decisions that no single team could have managed in isolation. The severity of underlying organ dysfunction illustrates that outcomes in spinal mucormycosis are determined as much by host factors as by antifungal selection. Both the most directly comparable prior case (Shah and Nene 2017, also a cirrhotic patient who expired at two weeks [8]) and the current report demonstrate that Child–Pugh Grade C liver disease represents a near‐prohibitive combination of immunosuppression and pharmacological constraint. Antifungal nephrotoxicity should be anticipated prospectively in patients with any preexisting renal impairment, and a substitution strategy involving posaconazole or isavuconazole should be preplanned rather than reactive.

This case carries the inherent limitations of a single case report, most notably the inability to draw generalizable conclusions about treatment efficacy. The absence of MIC data and incomplete laboratory documentation constrain the pharmacological analysis. The development of rapid molecular diagnostics capable of identifying Mucorales directly from tissue within hours, rather than the multiday culture timelines, represents the most impactful near‐term advance for this condition [1]. International registry development, analogous to the FungiScope registry for rare mold infections, would provide the systematic outcome data needed to build an evidence base for ultra‐rare infections such as spinal mucormycosis [3].

## 4. Conclusion

This report documents the 12th recorded case of spinal mucormycosis and the first in a patient with Child–Pugh Grade C hepatic cirrhosis as the primary immunocompromising condition. In tuberculosis‐endemic regions, mucormycosis must be included in the differential diagnosis of spondylodiscitis in immunocompromised patients, particularly when neurological deterioration continues despite empirical ATT. Tissue diagnosis is most efficiently achieved by combining surgical decompression with intraoperative biopsy when neurological urgency is present. Concurrent hepatic and renal impairment substantially narrows the antifungal therapeutic window and worsens prognosis. A coordinated multidisciplinary approach is the single most important factor in optimizing the chance of a favorable outcome in this rare and life‐threatening infection.

## Author Contributions

The following contributions are declared in accordance with the CRediT (Contributor Roles Taxonomy) authorship framework. Kanav Gupta: conceptualization, investigation, and writing–original draft. Kashif Qureshi: writing–review and editing. Sonal Gupta: writing–review and editing and supervision. Rakesh Dua: writing–review and editing and supervision. Shivanya Singh: writing–review and editing. Shaurya Darbari: writing–review and editing. Bipin Chaurasia: conceptualization, writing–review and editing, and supervision. Kivanc Yangi: writing–review and editing.

## Funding

This research received no external funding.

## Disclosure

All authors have read, reviewed, and approved the final manuscript and agree to be accountable for all aspects of the work.

## Ethics Statement

No formal institutional ethical approval was required for this case report. Written informed consent was obtained from the patient prior to initiation of this manuscript, as documented in the clinical records.

## Consent

Please see the Ethics Statement.

## Conflicts of Interest

The authors declare no conflicts of interest.

## Patient Perspective

A patient perspective could not be obtained as the patient did not survive the index hospitalization.

## Supporting Information

Additional supporting information can be found online in the Supporting Information section.

## Supporting information


**Supporting Information** The authors confirm that this case report was prepared in accordance with the CARE (CAse REport) 2013 guidelines. The completed CARE checklist is provided as a supporting file alongside this submission.

## Data Availability

All data relevant to this case report are contained within the manuscript and its supporting information. Additional data may be available from the corresponding author upon reasonable request.
