# Supplementary material for: Lumbar Spine Mucormycosis Mimicking Pott’s Spine: A Case Report
Source: Case Rep Infect Dis. 2026 Jun 8;2026:1299422. doi: 10.1155/crdi/1299422 (PMC13244248; doi:10.1155/crdi/1299422)
Supplement: Supplementary file 1 — Supporting Information The authors confirm that this case report was prepared in accordance with the CARE (CAse REport) 2013 guidelines. The completed CARE checklist is provided as a supporting file alongside this submission. [file CRDI-2026-1299422-s001.docx]

**CARE Checklist of Information to Include When Writing a Case Report**

*Manuscript: Lumbar Spine Mucormycosis Mimicking Pott's Spine: A Case Report*

| **Topic** | **Item** | **Checklist Item Description** | **Reported on Section / Line** |
| --- | --- | --- | --- |
| **Title** | **1** | The diagnosis or intervention of primary focus followed by the words "case report" | Title page |
| **Key Words** | **2** | 2 to 5 key words that identify diagnoses or interventions in this case report, including "case report" | Abstract section, Keywords line (6 keywords provided, including "case report") |
| **Abstract (no references)** | **3a** | Introduction: What is unique about this case and what does it add to the scientific literature? | Abstract, Background paragraph |
|  | **3b** | Main symptoms and/or important clinical findings | Abstract, Case Presentation paragraph (bilateral lower limb weakness, spondylodiscitis, epidural abscess) |
|  | **3c** | The main diagnoses, therapeutic interventions, and outcomes | Abstract, Case Presentation paragraph (Mucorales on histopath; liposomal AmB + posaconazole; died POD 12) |
|  | **3d** | Conclusion — What is the main "take-away" lesson(s) from this case? | Abstract, Conclusion paragraph (12th case; TB-endemic diagnostic challenge; multidisciplinary approach) |
| **Introduction** | **4** | One or two paragraphs summarizing why this case is unique (may include references) | Introduction section, paragraphs 1-4 (spinal mucormycosis rarity; first Child-Pugh C case; India TB-endemic context) |
| **Patient Information** | **5a** | De-identified patient specific information | Case Presentation, paragraph 1 (54-year-old male; no identifying information included) |
|  | **5b** | Primary concerns and symptoms of the patient | Case Presentation, paragraphs 1-2 (mechanical low back pain; bilateral lower limb weakness; urinary retention) |
|  | **5c** | Medical, family, and psycho-social history including relevant genetic information | **Medical history:** Case Presentation, paragraph 1 (CLD Child-Pugh C; DM; hypothyroidism; thrombocytopenia; portal HTN; ascites; prior oesophageal varices treatment June 2023). No family, psychosocial history was obtained from the patient. |
|  | **5d** | Relevant past interventions with outcomes | Case Presentation, paragraph 1 (Treatment for grade 3 oesophageal varices, June 2023; ATT initiated at first presentation) |
| **Clinical Findings** | **6** | Describe significant physical examination (PE) and important clinical findings | Case Presentation, paragraph 2 (3/5 power left LL; 4/5 power right LL; absent rectal tone; lumbar/dorsal tenderness; MRI findings) |
| **Timeline** | **7** | Historical and current information from this episode of care organized as a timeline | Table 2 (Clinical Timeline) (16-row table: initial presentation through death POD 12) |
| **Diagnostic Assessment** | **8a** | Diagnostic testing (such as PE, laboratory testing, imaging, surveys) | Case Presentation, paragraphs 2-5 (MRI lumbar spine x2; CT-guided biopsy considered; KOH mount histopathology; real-time PCR submitted) |
|  | **8b** | Diagnostic challenges (such as access to testing, financial, or cultural) | Case Presentation, paragraphs 3-4 (CT-guided biopsy deferred: inadequate for neural decompression + high hepatic operative risk; MDT meeting documented) |
|  | **8c** | Diagnosis (including other diagnoses considered) | Case Presentation, paragraphs 1, 4-5 (Initial diagnosis: suspected Pott's spine/tubercular spondylodiscitis; final diagnosis: spinal mucormycosis — Mucorales species on histopathology) |
|  | **8d** | Prognosis (such as staging in oncology) where applicable | Discussion, paragraphs 2 and 4 (Mortality >50% in immunocompromised patients; hepatorenal dysfunction severely constrains antifungal options) |
| **Therapeutic Intervention** | **9a** | Types of therapeutic intervention (such as pharmacologic, surgical, preventive, self-care) | Case Presentation, paragraphs 4-5 (Surgical: L3 laminectomy + L3-L4 epidural abscess drainage; Pharmacologic: liposomal amphotericin B; posaconazole; Supportive: ICU monitoring; paracentesis; electrolyte management) |
|  | **9b** | Administration of therapeutic intervention (such as dosage, strength, duration) | Case Presentation, paragraphs 5-6 (Liposomal AmB: 5 mg/kg/day IV; posaconazole: oral; total antifungal exposure: <12 days before death on POD 12) |
|  | **9c** | Changes in therapeutic intervention (with rationale) | Case Presentation, paragraph 6 (AmB withheld POD 3: nephrotoxicity + hypomagnesaemia; posaconazole commenced POD 3; AmB restarted ~POD 6: renal function stabilised; AmB withheld again: progressive bilirubin rise and hepatic decompensation) |
| **Follow-up and Outcomes** | **10a** | Clinician and patient-assessed outcomes (if available) | Case Presentation, paragraph 6 (Initial neurological improvement noted; death on POD 12 from multiorgan failure) |
|  | **10b** | Important follow-up diagnostic and other test results | Case Presentation, paragraph 6 (Serial renal profile; serum bilirubin trend; ascitic fluid assessment; electrolytes post-AmB; |
|  | **10c** | Intervention adherence and tolerability (How was this assessed?) | Case Presentation, paragraph 6 (Tolerability assessed via serial renal profile and LFTs; nephrotoxicity confirmed POD 3; hepatic decompensation confirmed ~POD 7-10) |
|  | **10d** | Adverse and unanticipated events | Case Presentation, paragraph 6 (Renal dysfunction + hypomagnesaemia POD 3; hepatic encephalopathy with sepsis; acute respiratory distress — mechanical ventilation declined by family; multiorgan failure POD 12) |
| **Discussion** | **11a** | A scientific discussion of the strengths AND limitations associated with this case report | Discussion, paragraph 6 (Limitations: single case report; absent MIC data; incomplete laboratory documentation; brevity of treatment course) |
|  | **11b** | Discussion of the relevant medical literature with references | Discussion, paragraphs 2-5; Table 1 (All 11-prior spinal mucormycosis cases reviewed; references [1]-[14]) |
|  | **11c** | The scientific rationale for any conclusions (including assessment of possible causes) | Discussion, paragraphs 1, 3, 4, 5 (Pathobiology of angioinvasion; diagnostic failure in TB-endemic context; antifungal pharmacology; hepatorenal constraints; MDT model) |
|  | **11d** | The primary "take-away" lessons of this case report (without references) in a one paragraph conclusion | Conclusion section (12th case; first Child-Pugh C case; TB-endemic differential; tissue diagnosis via decompressive surgery; antifungal constraints; MDT model) |
| **Patient Perspective** | **12** | The patient should share their perspective in one to two paragraphs on the treatment(s) they received | N/A since patient expired POD 12. |
| **Informed Consent** | **13** | Did the patient give informed consent? Please provide if requested | **YES.** Written informed consent obtained from patient prior to manuscript preparation. See Ethics Statement and Patient Consent section. |

*CARE 2013 guidelines: www.care-statement.org*
